# Supplementary material for: Hepatic bile acids and bile acid-related gene expression in pregnant and lactating rats
Source: PeerJ. 2013 Aug 27;1:e143. doi: 10.7717/peerj.143 (PMC3757468; doi:10.7717/peerj.143)
Supplement: Table S1 [file peerj-01-143-s001.docx]

**Supplementary Table 1: Primer sequences in real-time PCR analysis.**

| Gene | Access | Forward | Reverse |
| --- | --- | --- | --- |
| G3PDH | NM_017008.4 | AACTTTGGCATTGTGGAAGG | GGATGCAGGGATGATGTTCT |
| *β-actin* | NM_031144 | AGCCATGTACGTAGCCATCC | ACCCTCATAGATGGGCACAG |
| *Cyp7a1* | NM_012942 | GAGAACGGGTTGATTCCGTA | AAAAACGTGACCATGCTTC |
| *Cyp8b1* | NM_031241 | cacgtagccagtaccaagca | ggtcctagcatcaccaagga |
| *Cyp27a1* | NM_178847 | tctggctacctgcacttcct | gtctaccccagccaagatca |
| *Cyp7b1* | NM_019183 | tcatccgtgaagtgcaagag | ggagcatcgaagacttctgg |
| FXR | NM_021745 | CGAGATGCCTGTGACAAAGA | GCAGACCACACACAGCTCAT |
| *SHP* | NM_053908 | TTATGTGTGAGGGTGGACGA | CCCGTCTTCTTGAAGTGCTC |
| *Esr-1* | NM_012689 | TCCggCACATgAgTAACAAA | TgAAgACgATgAgCATCCAg |
| *PPAR-α* | NM_013196 | gagaccctcggggatcttag | tgtgtcctgagcttgaccag |
| *Ntcp* | NM_017047 | CACAACgTATCAgCCCCTTT | ATgCTAAgCgCCTTgTCTgT |
| *Bsep* | NM_031760 | CCACCAgAACATgACAAACg | CCCAgTgATgACCCATAACC |
| Mrp3 | NM_080581 | ccagacctcacaccctgttt | cgtcttgagcctggataagc |
| Mrp4 | NM_133411 | tgaagcaactgcaaatgtgg | agtgcactgggcaaacttct |
| *Oatp1* | NM_017111 | GGATGTAGCTGAGGCAGAGG | CAGCTCCCAGTGGCATTTAT |
| *Oatp2* | NM_0131906 | CCTAGGCATAGGCATTTGGA | TCAACCAAAGCACAAAGCAG |
| *Oatp4* | NM_031650 | AACATGCTTCGTGGGATAGG | CATGGAAGTGTGCCCTTCTT |
| *Abcg2* | NM_181381 | GAAAGACCCACGGGGATTAT | CCCATCACAACGTCATCTTG |
